# Effect of Antiplatelet Agents on Abdominal Aortic Aneurysm Process: A Systematic Review and Meta-Analysis

**DOI:** 10.31083/j.rcm2412357

**Published:** 2023-12-19

**Authors:** Yang Yang, Chang Li, Zhi-Yuan Wu, Zuo-Guan Chen, Yong-Peng Diao, Yong-Jun Li

**Affiliations:** ^1^Department of Vascular Surgery, Beijing Hospital, National Center of Gerontology; Institute of Geriatric Medicine, Chinese Academy of Medical Sciences, 100010 Beijing, China; ^2^Institute of Geriatric Medicine, Chinese Academy of Medical Science, 100010 Beijing, China; ^3^Peking University Health Science Center, 100010 Beijing, China; ^4^Peking Union Medical College, Chinese Academy of Medical Science, 100010 Beijing, China

**Keywords:** abdominal aortic aneurysm, antiplatelet agents, aspirin, endovascular aortic repair, meta-analysis

## Abstract

**Background:**

This systematic review and meta-analysis aims to investigate whether antiplatelet agents are associated with the reduction, expansion, and rupture of abdominal aortic aneurysm (AAA).

**Methods:**

A thorough exploration was conducted on four prominent databases, namely EMBASE, Ovid, PubMed, and Scopus, to identify studies that reported the influence of antiplatelet agents on the sac development of AAA. The assessment was carried out until March 2023. R software v.4.1 was used for statistical analysis.

**Results:**

After reviewing 13 publications which included a total of 5392 patients (1446 in the antiplatelet group and 2540 in the control group), a meta-analysis was conducted. The results of the analysis revealed that there was no significant difference in the annual growth rate of AAA diameter between those who received antiplatelet agents and those who did not (mean difference (MD) = –0.04, 95% CI = [–0.37, 0.30]; heterogeneity: *p <* 0.01, $I^{2}$ = 91%, τ^2^ = 0.1278). Additionally, there was no difference in the number of patients who experienced aneurysm diameter expansion between the two groups, significantly (odds ratio (OR) = 0.96, 95% CI = [0.41, 2.25]; heterogeneity: *p <* 0.01, $I^{2}$ = 78%, τ^2^ = 0.5849).

**Conclusions:**

Antiplatelet agents do not affect AAA’s reduction, expansion, or rupture. There is no benefit to AAA patients taking antiplatelet agents for the purpose of slowing down growth rates of sac diameter.

## 1. Introduction

Abdominal aortic aneurysm (AAA) is a specific form of atherothrombotic disease, which is usually characterized by dilated abdominal aorta greater than 30 mm or exceeding 50% the normal aortic diameter [1]. Endovascular repair, open surgery, and various medications such as statins, aspirin, and warfarin were used to slow the expansion rate and even regress sac volume [2, 3, 4, 5]. However, the result of using the above methods for restricting the expansion rate on AAA was still uncertain. The most recent guideline from the European Society for Vascular Surgery (ESVS) recommends the use of antiplatelet agents for patients undergoing AAA surgery, however this is without any evidence for the potential impact of antiplatelet agents on sac volume [6]. To further clarify the role of antiplatelet agents in the treatment of AAA, we performed a systematic review and meta-analysis to investigate whether antiplatelet agents are associated with the reduction, expansion, and rupture of sac capsules.

## 2. Methods

### 2.1 Study Design

We registered the analysis protocol under the registration number CRD42022326589, on the International Prospective Register of Systematic Reviews (PROSPERO). The analysis followed the guidelines provided in the Preferred Reporting of Systematic Reviews and Meta-Analysis (PRISMA) statement [7]. The primary objective of the analysis was to investigate the impact of antiplatelet treatment on sac volume in patients with AAA. To select relevant articles, the P.I.C.O. (patient: patients with AAA; intervention: antiplatelet treatment; comparison: antiplatelet medication vs. placebo or other medications; outcome: sac regression or expansion, and rupture, among others) model was utilized [8].

### 2.2 Search Strategy

For this study, a search was conducted across four databases, EMBASE, Ovid, PubMed, and Scopus. The literature search strategy utilized the following keywords: (“aspirin” OR “clopidogrel” OR “antiplatelet”) AND (“abdominal aortic aneurysm” OR “aortic aneurysm repair” OR “aortic diameter” OR “growth rate”). The search was conducted up until March 2023. In addition, references for all included literature and for similar Meta-analyses were searched.

### 2.3 Inclusion and Exclusion Criteria

The inclusion criteria for this study were as follows: (1) reported results for cohorts of more than 10 patients receiving antiplatelet agents with at least one imaging data, and (2) compared the sac change of AAA patients receiving antiplatelet agents with that of patients receiving placebo or other agents without antiplatelet function. Studies that did not meet the following exclusion criteria were not considered: (1) case reports, meetings, and literature reviews, (2) studies that referred to biomarkers of AAA patients unless they included radiographic features of the sac volume, (3) articles with inadequate data (less than 25% of predefined variables extractable), and (4) studies reporting on the same population of patients. In the latter case, only the latest report was included unless the outcomes were mutually exclusive.

### 2.4 Literature Screening and Data Extraction

**Fig. 1.**
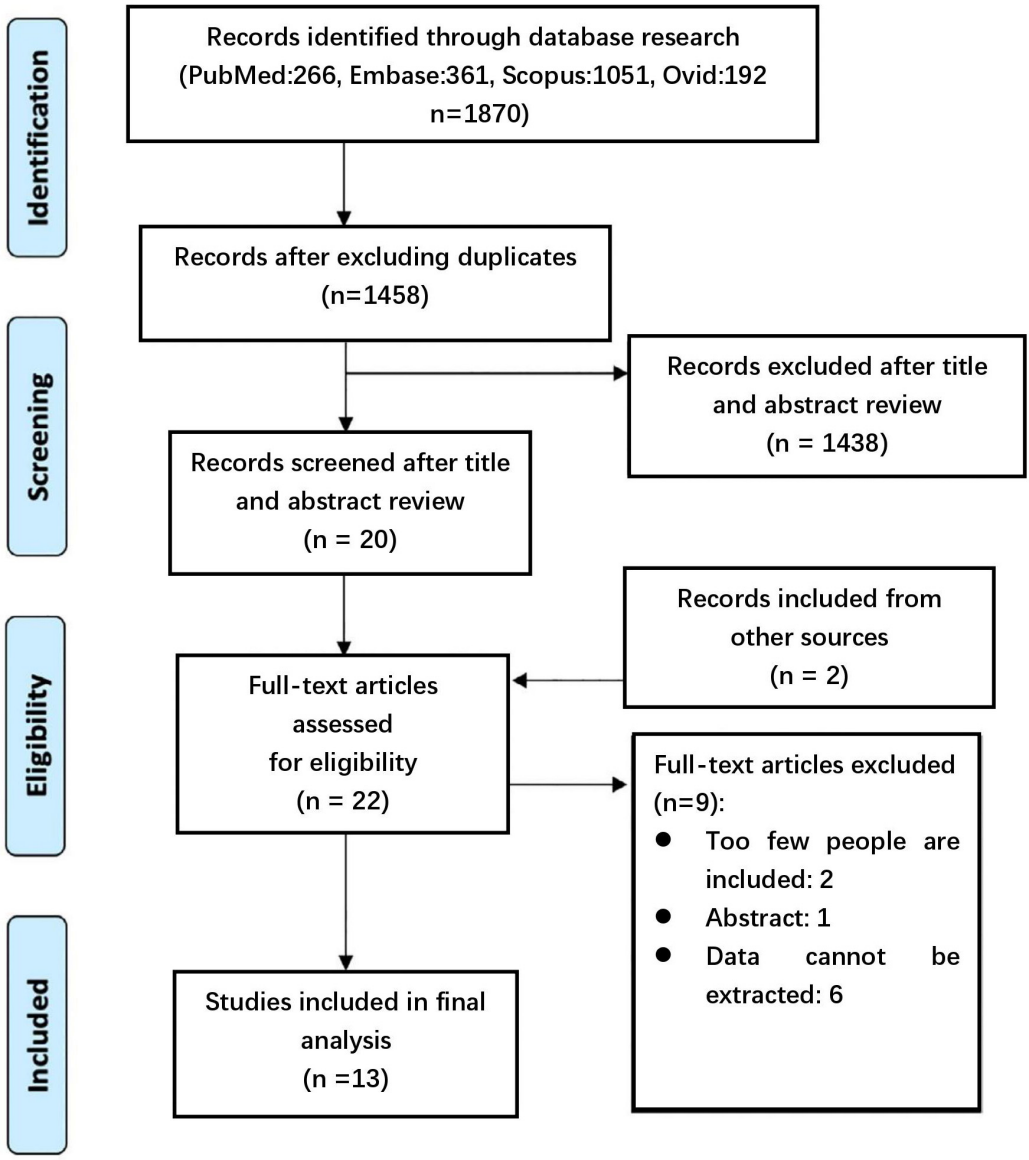
**Flow diagram adhering to the Preferred Reporting Items for Systematic Review and Meta-Analysis guidelines to illustrate the search and selection process during the initial stages of our review**.

Duplicate citations were removed, and an independent reviewer was responsible for reviewing all titles and abstracts. Full-text versions of studies that met the inclusion criteria were obtained, and data extraction was performed by another independent reviewer. In cases where a consensus could not be reached between the two reviewers, a third reviewer was consulted to assist with the re-review of the full text of the article (Fig. 1). The extraction of data was performed by the first author and independently verified by the co-authors using a standardized data collection. Data collected included first author, publication year, study design, sample size, interventions, follow-up year, and outcome data (sac diameter reduction or expansion, and rupture).

### 2.5 Risk of Bias Assessment

The Cochrane Risk of Bias tool was used to assess the quality of included randomized controlled studies, including selection bias, performance bias, detection bias, attrition bias, reporting bias, and other biases [9]. Non-randomized controlled studies were assessed by the Newcastle-Ottawa scale (NOS) for selection bias in the antiplatelet drug and control groups, comparability bias in the two cohorts, and outcome assessment bias [10].

### 2.6 Statistical Analysis

The continuous variables were compared between the two groups using *t*-test and the categorical variables were compared using the χ^2^-test. The random effects model was used to evaluate the results. The proportion was compared between the two groups to see if there was an overlap of 95% confidence intervals (CI) to assess statistical significance. Statistical analysis was performed using R software (Foundation for Statistical Computing, Vienna, Austria. URL http://www.R-project.org/. version 4.1).

## 3. Results

### 3.1 Literature Screening

We reviewed a total of 1872 references published within the predefined time frame of the analysis. Following a thorough examination of 22 articles, we ultimately included 13 studies that reported on the effects of antiplatelet agents on AAA progression in a total of 5392 patients (Table 1, Ref. [5, 11, 12, 13, 14, 15, 16, 17, 18, 19, 20, 21, 22]). Three of them were randomized controlled clinical studies, two were prospective studies, and eight were retrospective studies. Eight studies reported on annual growth rate of sac diameter after taking antiplatelet agents; four reported on the number of cases of change in aneurysm diameter after taking antiplatelet agents; and one reported on aneurysm rupture after taking antiplatelet agents in patients. In terms of the following baseline characteristics: gender, smoking, and basic disease (including hypertension, diabetes mellitus, chronic obstructive pulmonary disease, and chronic renal insufficiency), there were no significant differences between the antiplatelet drug group and the control group (*p* > 0.05). However, there was a significantly higher proportion of patients with coronary artery disease in the antiplatelet drug group as compared to the control group (χ^2^ = 8.286, *p* = 0.004 < 0.05) (Table 2).

**Table 1.** **Basic characteristics of included literature and quality assessment of non-randomized controlled studies**.

Research	Type of research	Year of publication	Duration	Drugs	Number of patients		Outcome measurement	Ending	NOS score
					Non-antiplatelet	Antiplatelet			
Lindholt *et al*. (diameter >40 mm) [11]	Randomized controlled study	2008	10 years	Low-dose aspirin	17	14	Aneurysm diameter growth rate (mean ± standard deviation, mm/year)	Unmedicated increase 2.92 ± 2.82; medicated increase 5.18 ± 2.41	NA.
Lindholt *et al*. (diameter <40 mm) [11]	Randomized controlled study	2008	10 years	Low-dose aspirin	69	48	Aneurysm diameter growth rate (mean ± standard deviation, mm/year)	Unmedicated increase 2.52 ± 2.06; medicated increase 2.23 ± 1.45	NA.
Wanhainen *et al*. [12]	Randomized controlled study	2020	1 year	Tegretol	69	67	Aneurysm diameter growth rate (mean ± standard deviation, mm/year)	The diameter of the sac increased by 1.8 ± 1.91 without medication; the diameter of the sac increased by 2.5 ± 2.30 with medication	NA.
Karlsson *et al*. [13]	Randomized controlled study	2009	18 months	Aspirin	110	101	Aneurysm diameter growth rate (mean ± standard deviation, mm/year)	Increase in sac diameter without medication 2.6 ± 7.22; increase in sac diameter with medication 1.8 ± 6.13	NA.
Aoki *et al*. [14]	Retrospective study	2011	6 months	Anti-platelet agents	34	23	Aneurysm diameter reduction rate (mean ± standard deviation, mm/year)	Decrease in sac diameter without medication 14.6 ± 11.8; decrease in sac diameter with medication 9.4 ± 11.0	8
Thompson *et al*. [15]	Retrospective study	2010	25 years	Anti-platelet agents	757	443	Aneurysm diameter growth rate (mean ± standard deviation, mm/year)	The diameter of the sac increased by 1.54 ± 0.15 without medication; the diameter of the sac increased by 1.35 ± 0.83 with medication	9
Ahmad *et al*. [16]	Retrospective study	2017	20 years	Aspirin	77	40	Aneurysm diameter growth rate (mean ± standard deviation, mm/year)	Increase in sac diameter without medication 2.4 ± 2.2; increase in sac diameter with medication 2.0 ± 2.2	8
Sweeting *et al*. [17]	Forward-Looking Research	2010	1.9 years	Anti-platelet agents	1200	501	Aneurysm diameter growth rate (mean ± standard deviation, mm/year)	The diameter of the sac increased by 2.76 ± 0.16 without medication; the diameter of the sac increased by 2.91 ± 0.16 with medication	7
Rasmussen *et al*. [18]	Forward-Looking Research	2014	1.78 years	Aspirin	207	209	Aneurysm diameter growth rate (mean ± standard deviation, mm/year)	The diameter of the sac increased by 3.01 ± 2.67 without medication; the diameter of the sac increased by 2.42 ± 2.46 with medication	7
Chen *et al*. [5]	Retrospective study	2013	4.01 years	Low-dose aspirin	110	118	Aneurysm rupture	Ruptured sac without medication 2; ruptured sac with medication 5	7
Morisaki *et al*. [19]	Retrospective study	2022	1 year	Anti-platelet agents	103	79	Reduction in sac diameter	Decrease in sac diameter without medication 39; decrease in sac diameter with medication 16	8
Balceniuk *et al*. [20]	Retrospective study	2018	NA.	Aspirin	43	223	Reduction in sac diameter	Reduction in sac diameter by drug administration OR: 3.327, 95% CI (1.409–7.857), *p* = 0.006	8
Marcos *et al*. [21]	Retrospective study	2017	41.5 months	Anti-platelet agents	12	66	Expansion in sac diameter	Increase in sac diameter without medication 4; increase in sac diameter with medication 21	8
Ferguson *et al*. [22]	Retrospective study	2010	5 years	Aspirin	289	363	Expansion in sac diameter	Increase in sac diameter by drug administration OR: 1.10, 95% CI (0.78–1.56), *p* = 0.575	9

NOS, Newcastle-Ottawa scale; OR, odds ratio. NA means data is not available.

**Table 2.** **Baseline patient characteristics**.

	Control group (n/n)	Anti-platelet agents group (n/n)	χ ^2^	*p*-value
Male	235/283 (0.83)	223/266 (0.84)	0.018	0.892
Smoking	157/259 (0.61)	117/210 (0.56)	0.955	0.329
High blood pressure	210/295 (0.71)	256/332 (0.77)	2.569	0.109
Diabetes	35/283 (0.12)	40/266 (0.15)	0.618	0.432
Coronary heart disease	45/122 (0.37)	100/184 (0.54)	8.286	0.004
Chronic obstructive pulmonary disease	50/192 (0.26)	66/253 (0.26)	0	1
Renal insufficiency	10/110 (0.09)	15/118 (0.13)	0.439	0.508

### 3.2 Literature quality assessment

**Fig. 2.**
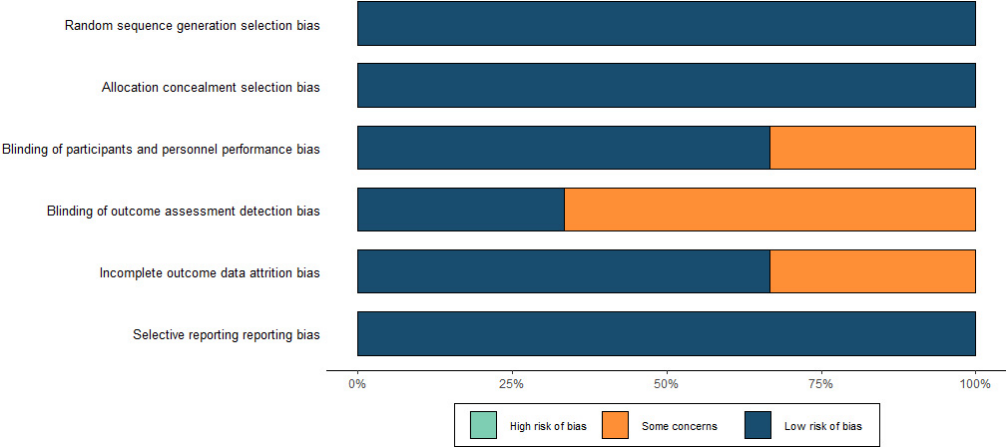
**Risk of bias**. The ROBINS-I checklist for randomized trials was used to assess the methodological quality and potential risk of bias. ROBINS-1, Risk of Bias In Non-Randomized Studies of Interventions.

Risk assessment using the Cochrane Risk Assessment Tool for all included randomized controlled clinical studies showed a low overall risk for these three studies [11, 12, 13] (Fig. 2). The quality of the literature for non-randomized controlled studies was assessed using the NOS scale and was high in all cases (Table 1).

### 3.3 Meta-Analysis Results

**Fig. 3.**
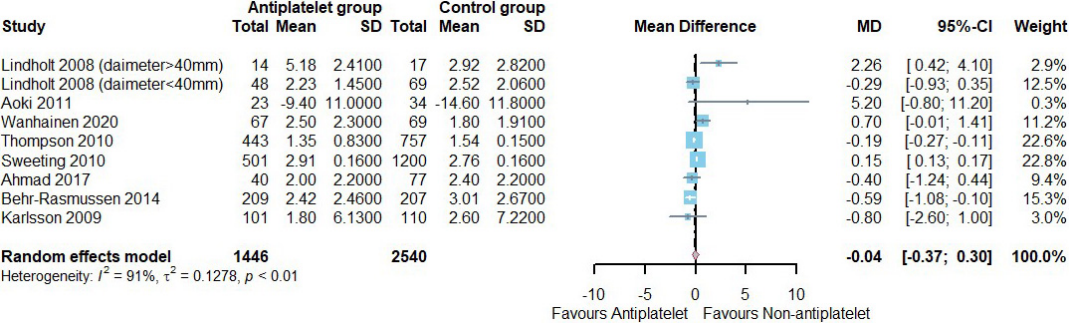
**Annual growth rate of AAA diameter (mm/year) in patients using antiplatelet agents vs. not using antiplatelet agents**. SD, standard deviation; MD, mean difference; CI, confidence interval; AAA, abdominal aortic aneurysm.

Of the total studies, the AAA growth rate was reported in eight studies (antiplatelet group: 1446, control group: 2540), including four with aspirin, one with ticagrelor, and three with any antiplatelet agents [11, 12, 13, 14, 15, 16, 17, 18]. Of all the literature, only one reported a reduction in AAA diameter with the use of antiplatelet agents (control group: 14.6 ± 11.8 mm/year vs. antiplatelet group: 9.4 ± 11.0 mm/year) [14]. We added a 16 mm/year diameter change based on all annual growth rates, and our meta-analysis did not reveal a statistically significant difference in the annual growth rate of AAA diameter with or without antiplatelet agents (mean difference (MD) = –0.04, 95% CI = [–0.37, 0.30]; heterogeneity: *p <* 0.01, $I^{2}$ = 91%, τ^2^ = 0.1278) (Fig. 3).

**Fig. 4.**
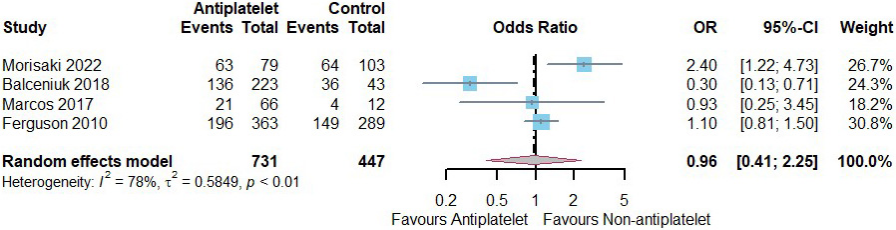
**Number of patients (n) who experienced aneurysm diameter expansion using antiplatelet agents vs. not using antiplatelet agents**. CI, confidence interval; OR, odds ratio.

Four studies (antiplatelet group: 731, control group: 447) reported on the number of cases of aneurysm diameter change after the use of antiplatelet agents, two with aspirin and two with any antiplatelet drug [19, 20, 21, 22]. Meta-analysis showed no significant difference between AAA diameter growth with or without antiplatelet agents (odds ratio (OR) = 0.96, 95% CI = [0.41, 2.25]; heterogeneity: *p <* 0.01; $I^{2}$ = 78%, τ^2^ = 0.5849) (Fig. 4).

Only one study described aneurysm rupture after the use of antiplatelet agents with a multifactorial Cox regression analysis showing that the risk of AAA rupture was not significantly altered with or without low-dose aspirin (hazard ratio (HR) = 1.019, 95% CI = [0.993, 1.044], *p* = 0.951) [5].

## 4. Discussion

The results of our meta-analysis suggest that antiplatelet agents do not affect AAA process, either in terms of sac expansion, reduction, or rupture. Although some of the findings hold opposite opinions, an increasing number of studies suggest that the effectiveness of antiplatelet agents in slowing the expansion of AAA may be due to the antiplatelet agents’ capacity to decrease platelet-derived cytokines expression and plasminogen activation, and to diminish the infiltration of platelets and macrophages within the vascular wall [11, 13, 22, 23]. Our findings may be different, as meta-analysis does not demonstrate any significant differences in growth rates in patients taking antiplatelet agents (MD = –0.04, 95% CI = [–0.37, 0.30]) (Fig. 3).

Unlike statins, which have been clearly demonstrated to slow AAA expansion and prevent rupture through anti-inflammatory and antioxidant effects and by reducing matrix metalloproteinase secretion [24, 25], the mechanism of antiplatelet agents on AAA remains to be investigated. Hofmann *et al*. [26] analyzed the effects of aspirin and therapeutic anticoagulants on mRNA and protein expression of heme oxygenase-1 (HO-1) in AAA patients. They showed that aspirin and therapeutic anticoagulants were not significantly associated with HO-1 expression (*p* > 0.05) and were unable to induce HO-1 gene overexpression to provide protection against oxidative stress cells. On the other hand, some researchers suggest that antiplatelet agents may indirectly slow down AAA expansion by inhibiting the progression of intraluminal thrombus (ILT) [23, 27]. ILT-induced AAA diameter growth and rupture are the results of a combination of mechanisms, including laminar-to-turbulent flow changes due to altered hemodynamics; anterior wall deposition due to asymmetric spatial distribution of ILT in the AAA capsule [23, 28, 29]. However, part of the studies conclude that the effect of antiplatelet agents on ILT is limited. Sagan *et al*. [30] and Gerasimidis *et al*. [31] concluded that aspirin-mediated antiplatelet antithrombotic effects do not have the desired effect and even increase mortality within 30 days due to the high fibrin content of ILT [32]. Slowing the growth rates of AAA diameter by inhibiting ILT formation and progression is controversial, which requires more prospective studies to demonstrate the role of antiplatelet agents in ILT and AAA.

Of note, a total of three included papers described a reduction in AAA aneurysms in patients who underwent Endovascular aneurysm repair (EVAR) of abdominal aorta with the use of antiplatelet agents. Aoki *et al*. [14] found that there was no significant difference in AAA diameter reduction in patients with or without antiplatelet agents after receiving EVAR (control group: 14.6 ± 11.8 mm/year vs. antiplatelet group: 9.4 ± 11.0 mm/year, *p* = 0.163), that AAA diameter reduction was mainly the result of receiving EVAR, and that after multifactorial regression analysis, multiple antiplatelet treatments significantly inhibited the diameter reduction of AAA (*p* = 0.422). After receiving EVAR, Morisaki *et al*. [19] reported a lower frequency of AAA diameter reduction in the antiplatelet group compared to the control group (37.9% vs. 20.3%); the results of Balceniuk *et al*. [20] concluded that the use of aspirin was an independent predictor of AAA sac reduction after receiving EVAR (OR = 3.327, 95% CI = [1.409, 7.857], *p* = 0.006). Since its introduction in 1991, EVAR has now become one of the main options for the treatment of AAA. Whether antiplatelet agents should be used after EVAR seems to be of more concern than drug therapy alone, and larger clinical trials are needed to demonstrate the effect of antiplatelet agents on AAA sac after receiving EVAR.

## 5. Conclusions

In summary, antiplatelet agents have no effect on the development of AAA, either in terms of AAA expansion, reduction or rupture, and there is no benefit to AAA patients taking antiplatelet agents for the purpose of slowing down the growth rates of sac diameter.

## Supplementary Material

Supplementary material associated with this article can be found, in the online version, at https://doi.org/10.31083/j.rcm2412357.
